# Study of the Percentage of Greenhouse Gas Emissions from Aviation in the EU-27 Countries by Applying Multiple-Criteria Statistical Methods

**DOI:** 10.3390/ijerph17113759

**Published:** 2020-05-26

**Authors:** Miriam Andrejiová, Anna Grincova, Daniela Marasová

**Affiliations:** 1 Faculty of Mechanical Engineering, Technical University of Kosice, 042 00 Kosice, Slovakia; miriam.andrejiova@tuke.sk; 2 Faculty of Electrical Engineering and Informatics, Technical University of Kosice, 042 00 Kosice, Slovakia; 3 Faculty of Mining, Ecology, Process Control and Geotechnology, Technical University of Kosice, 042 00 Kosice, Slovakia daniela.marasova@tuke.sk

**Keywords:** air quality, greenhouse gas emissions, aviation, EU-27, PCA method, cluster analysis

## Abstract

The transport sector, including air transport, represents an important source of air pollution. The present article deals with the current situation regarding greenhouse gas emissions in the air in 27 European Union (EU-27) member states. Every member state is characterized by selected parameters that determine the unique nature of a particular country (e.g., population, area, life expectancy, gross domestic product (GDP) per capita, etc.). In addition to these parameters, there were also other parameters which were monitored as they characterize the amount of greenhouse gas emissions and the impact of aviation on these emissions. The main purpose of the article is to compare the European Union member states on the basis of 15 examined parameters. The identification of similarities between the EU-27 member states with regard to the selected parameters was carried out by applying principal component analysis (PCA) and hierarchical cluster analysis. The average linkage method was applied to create a dendrogram representing the similarities between the examined member states. The value of the cophenetic correlation coefficient CC = 0.923 confirmed the correct application of the average linkage method. The cluster analysis outputs were five similarity-based homogeneous groups (clusters) into which the 27 member states were divided on the basis of the examined variables.

## 1. Introduction

Every year, more than 400,000 Europeans die early due to poor air quality, and many others suffer from respiratory and cardiovascular diseases caused by air pollution [1]. From an economic point of view, poor air quality represents a cost of more than 20 billion euros per year for the European Community, as it increases healthcare costs, reduces the productivity of the labor force, and damages the soil, crops, forests, lakes and rivers [1].

Low air quality and climate change are closely related to elevated greenhouse gas (GHG) concentrations in the atmosphere (carbon dioxide (CO_2_), methane (CH_4_), nitrous oxide (N_2_O), chlorofluorocarbons (CFCs), hydrofluorocarbons (HFCs), perfluorocarbons (PFCs), sulfur hexafluoride (SF_6_) and nitrogen trifluoride (NF_3_)).

Excessive greenhouse gas emissions result in global climate change, which may directly (e.g., changes in weather conditions, extremely high temperatures, droughts, floods, etc.) or indirectly (e.g., impaired quality of the air, water, soil, changes in agriculture and living conditions, etc.) affect both human health and various ecosystems.

According to [2], greenhouse gas emissions reached a peak value in the late 1980s. In the years 1990–1994, this value decreased by approximately 28%, and since 1995 GHG emissions have stayed at approximately the same level. In 2017, the total greenhouse gas emissions in the EU-27 decreased by almost 27% compared to the values observed in 1990; this represents an absolute reduction of 1240 million tonnes of CO_2_ equivalents [3]. In order to maintain an acceptable air pollution level, certain international conventions, protocols and guidelines were concluded and adopted [4,5,6,7]. Their role is to ensure climate protection and achieve the stabilization of greenhouse gas concentrations at levels that do not create interference with the climate system. The main objective of the European Union (EU) policy on the protection of the environment and public health is to provide comparable information on the health and the state of individual environment components in the EU.

Despite the efforts aimed at reducing greenhouse gas emissions, and in view of the existing standards, the majority of the EU population does not live in a healthy environment [8]. Exposure to excessive air pollution may cause a number of negative health-related consequences [9]. In many studies, certain correlations were observed between daily changes in the air pollution and the increased occurrence of cardiorespiratory diseases [10,11] or cardiorespiratory mortality [12,13].

There are various sources of air pollution, i.e., the sources of emissions. The important ones include air transport, which significantly affects the greenhouse gas emissions. According to [3], in 2017, fuel combustion and fugitive emissions from fuels, without transport, represented 54% of the total greenhouse gas emissions in the EU-27. In 1990, this source segment was even more dominant (62%). Fuel combustion in transport, including international aviation, was the second largest source of emissions in 2017, representing 25% of the total. When compared to the year 1990, there was an 8% increase. Industrial processes and product use represented 8%. Greenhouse gas emissions from agriculture represented a 10% contribution to the total greenhouse gas emissions in the EU while the lowest contribution to the total emissions was observed in the waste management sector.

The ecological effects of the transport sector on the environment are negative and often irreversible [14]. That is why increasing attention is being paid to transport efficiency in terms of energy consumption and the production of greenhouse gases. Article [15] presented a verified method of calculating the energy and emission efficiency of the transport system, which is based on the applicable European standards.

The percentage of all transport sector contributions to the production of greenhouse gas emissions in the EU-27 in the period from 1990 to 2017 increased by 10% [3]. According to the latest summaries of the European Committee for the period of 2014–2024, the current aviation sector contributes to the production of approximately 7% of all emissions produced by the transport industry and approximately 2% of the total anthropogenic emissions of carbon dioxide. However, this share is increasing extremely fast as aircraft operations increase. According to the European Aviation Environmental Report 2019 [16], greenhouse gas emissions from aviation activities have more than doubled over the last two decades. Therefore, prior to the 25th Conference of the Parties (COP25) in Madrid (December 2019), the European Parliament adopted a resolution requesting that the EU adopt substantial measures aimed at reducing aviation emissions.

Article [17] presents information on the policies and objectives of international aviation organizations (particularly the Advisory Council for Aeronautics Research in Europe (ACARE) and the International Air Transport Association (IATA)), as well as the European Committee, in the reduction of aviation emissions, including basic information on the EU system for emission trading and its relationship to the integration of the emissions into air transport and the ambitious goals determined in the document titled European Aviation Vision 2050, which deals with the environmental impact of aviation. Polishchuk et al. [18] developed and applied fuzzy modeling to information technology for making quantitative estimates of the emerging environmental projects in the air transport segment.

Koscak et al. [19] focused their work on the creation of an algorithm for airport winter maintenance modeling. The purpose of the algorithm is to reduce maintenance times as well as the negative environmental impact of winter maintenance.

The general efforts aimed at maintaining air quality represent a topic studied by many experts (scientists and organizations). Jakubiak [20] analyzed the methods of reducing the environmental impact of aviation. The inclusion of aircraft operators in the emissions trading system of the European Union was also introduced. Cerro et al. [21] presented the results of air pollution measurements at a regional background site in the Balearic Islands. They also monitored the air pollution sources related to the transport sector. Mocerino et al. [22] proposed an optimal network for monitoring air quality, which facilitated the quantification of the pollutant emissions produced by maritime transport. Their mobile monitoring proved to be a useful technique for characterizing the spatial variability of air pollution in urban regions and the concentration gradients of pollutants from particular sources [23]. In their case study, Dong et al. [24] used a model for assessing the population relative risk of air pollution exposure and methods for determining the air pollution concentration with the goal of identifying the optimal method of assessing the risk of the population’s exposure to sulfur oxides (SO2).

Vichi et al. [25] conducted a survey on the impact of civil aviation on air quality. The 5-year research project was carried out at two international airports in central Italy. Paper [26] presents the approaches aimed at the identification and quantification of an airport’s contribution to the NOx concentrations near the airport. The authors of the paper [27] analyzed the data on emissions from global commercial aviation for the period of 2004–2006. The impact of long-distance passenger transport (mainly road and air transport) on greenhouse gas emissions and the related costs were described by the authors of the paper [28].

According to [29], aircraft emissions contribute to climate change (ozone depletion and greenhouse effect), acidification and disturbances (local air pollution and smells). The main factor contributing to this air pollution is civil aviation, but military aviation also plays a certain role.

The methods of multidimensional statistical analysis are used in many research areas, such as medicine, biology, psychology, technical sciences, etc. A multidimensional statistical analysis was applied within the examination of correlations between the N2O emissions and the online operational variables (concentrations of dissolved oxygen and nitrogen, temperature and flow velocity) in the biological removal of nitrogen from wastewater [30]. Belandi et al. [31] applied principal component analysis and the clustering method to identify the critical processes related to N_2_O emissions reduction. The authors of [32] used multidimensional statistical methods (factor, cluster, discriminant and regression analysis) to identify the impact of a tropical reservoir on the production of greenhouse gases. Ni et al. [33] combined the principal component analysis (PCA) method and the deep belief network (DBN) method in their evaluation of civil aviation safety. On the basis of the PCA method, a complex approach to the assessment of the aviation maintenance support ability was developed [34]. A combination of the PCA method and artificial neural networks (ANN) was also applied to identify the pollution sources at ten locations in Malaysia [35]. When comparing the greenhouse gas emissions in 24 Asian countries, the authors of the paper [36] used the PCA method and a cluster analysis. The results of the study were used to prepare procedures for reducing greenhouse gas emissions in various industries (energy, industrial processes, agriculture, waste, land use changes, and forestry and bunker fuels).

## 2. Material and Methods

### 2.1. Data Sources

**Figure 1:**
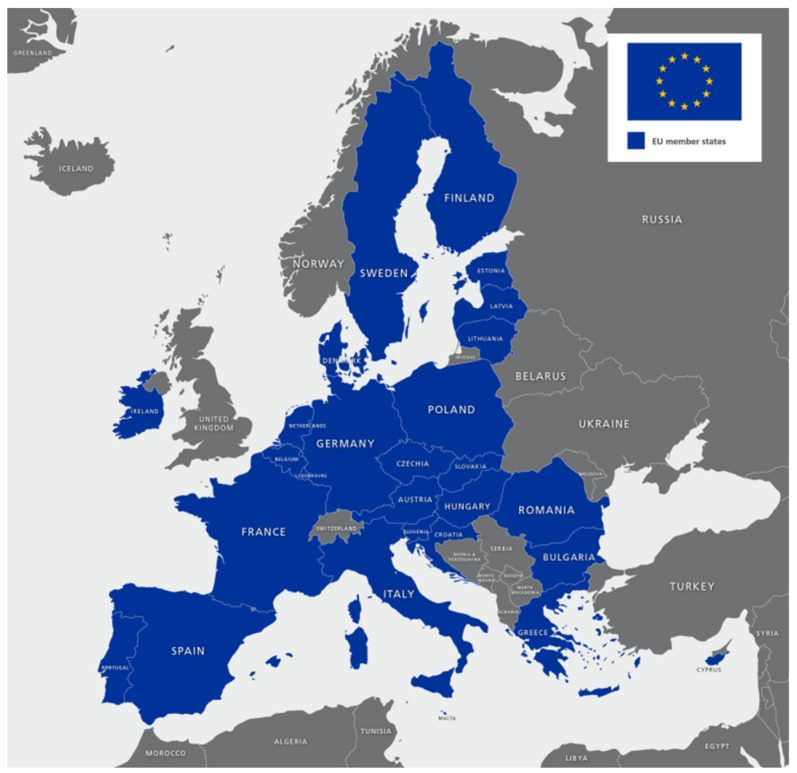
Map of the European Union (EU, countries shown in blue) [37].

The European Union currently includes 27 member states (Figure 1), with the total area of 4.4 million km^2^ and approximately 437 million inhabitants. The main purpose of the European Union is to provide Europe with remarkable economic growth, a competitive economy and improvement of environment quality.

In order to compare the 27 European Union (EU-27) member states, we selected several parameters (A1–A15). Each country is characterized by its population (A1) and area in km2 (A2). The gross domestic product per capita (A3) represents one of the key indicators of the quality of life. Healthy life years (A4) and life expectancy (A5) represent important indicators of the public health in each country.

Concerning the purpose of this article—monitoring the impact of aviation on greenhouse gas emissions—more parameters were determined for the countries in question: the number of commercial airports with more than 15,000 passengers per year (A6), the total number of aviation passengers (A7), the total number of aviation goods (A8), the total greenhouse gas emissions in thousand tonnes (A9) and the amount of aviation greenhouse gases in thousand tonnes (A10). In addition to the total greenhouse gas emissions in thousand tonnes, the monitoring also focused on the amounts of individual aviation greenhouse gas emissions in thousand tonnes (CO2–A11, N2O–A12, CH4–A13, HFC–A14).

Public health is also endangered by solid particles in the air. The monitored parameters were therefore supplemented with the total amount of PM2.5 particulate matter in the air (A15) in tonnes.

### 2.2. Statistical Methods

The analysis of the greenhouse gas emissions in the air was carried out applying the basic statistical methods and multidimensional statistical methods: principal component analysis (PCA) and a cluster analysis.

#### 2.2.1. PCA Method

Principal component analysis belongs to the elementary methods of a multidimensional analysis. The purpose thereof is to reduce the number of correlated variables to the principal components with the least possible loss of information [38,39]. The principal components are formed as linear combinations of the original (input) variables.

Let $A_{i}$, i=1,2,⋯,k be *k* examined variables. Each of the new variables (principal components) $Y_{i}$, i=1,2,⋯,k is a linear combination of the original *k* variables

(1) $$Y_{i}=l_{i1}A_{1}+l_{i2}A_{2}+\cdots +l_{ik}A_{k},\;\mathrm{for}\;i=1,2,\cdots ,k,$$

where $l_{ij}$, i,j=1,2,⋯,k are the coefficients (weights) subjected to the following condition:

(2) $$\sum_{i=1}^{k}l_{ij}^{2}=1\;\mathrm{for}\;=1,2,\cdots ,k.\;$$

Vector $l_{i}$, $l_{i}=\left(l_{i1},l_{i2},\;\cdots ,l_{ik}\right)$ is the eigenvector of the correlation matrix and its components are identified as the normed solutions of the system of equations

(3) $$\left(\sum_{x}-\lambda_{i}I\right)l_{i}=0,$$

where $\sum_{x}$ is the covariance matrix of the original variables, ***I*** is the unit matrix, ***0*** is the zero matrix and $\lambda_{i}$ is the solution of the following equation:

(4) $$\left|C-\lambda I\right|=0.$$

The equation is referred to as the characteristic equation of matrix $\sum_{x}$ with the eigenvalues being the solutions. For each eigenvalue $\lambda_{i}$ there is an eigenvector $l_{i}$.

The first principal component $Y_{1}$ accounts for the largest possible variance of the original values; the contributions of the other principal components to the variance are always lower. These new components express almost the entire variability of the original variables and there are no correlations between them.

An appropriate number of principal components may be determined applying several methods. In practice, the Kaiser–Guttman rule is applied; it takes into consideration all the eigenvalues higher than 1. Another rule recommends considering only those principal components which explain 70% to 90% of the cumulative variance [39].

The PCA method may also be used for the detection of outliers which must be, in some cases, excluded from the subsequent analysis. This method is a useful tool for the categorization of objects into clusters.

#### 2.2.2. Cluster Analysis

A cluster analysis is a statistical method that belongs to multidimensional statistical methods. Its purpose is to group the monitored objects into certain similar, homogenous groups—clusters. The graphical representation of these groups is a dendrogram. Depending on the clustering method, the procedures are divided into hierarchical and nonhierarchical methods [40,41].

The use of hierarchic clustering methods is based on the gradual grouping of objects, from the most similar ones to the most different ones. At the first level, each object forms a single cluster. In the following step, the most similar clusters are again grouped. At the last level, all objects are grouped into a single cluster.

As for the hierarchical methods, the ones that are most frequently used include the nearest neighbor method, the furthest neighbor method, the average linkage method, the centroid method, the median method and Ward’s method.

The clustering is performed using various degrees of similarity between the objects, including the distance between two objects. Let us have *n* objects, each characterized with *k* variables. The distance may be expressed using the Euclidean distance:

(5) $$d_{ij}=\sqrt{\sum_{i=1}^{k}{\left(X_{ik}-X_{jk}\right)}^{2},}$$

where $X_{ik}$ is the value of the k^th^ variable for the i^th^ object, and $X_{jk}$ is the value of the k^th^ variable for the j^th^ object.

Within the first clustering, the matrix of distances ***D*** between two objects (each object represented a single cluster) is calculated. The matrix ***D*** is then used to find two clusters with the minimum distance, which are grouped into one cluster. This process is repeated until all the objects are grouped into a single cluster. When the average distance method is applied, two clusters are combined into a new cluster if they exhibit the average minimum distance.

The graphical output of the clustering is a hierarchic tree, referred to as a dendrogram. All the data were evaluated and the results were obtained using the R-package program.

## 3. Result and Discussion

The research was carried out with the objective to:

- analyze aviation’s contribution to the production of greenhouse gas emissions in the air of the EU-27 member states;
- compare the EU-27 member states in terms of the impact of aviation on greenhouse gas emissions and other parameters that characterize a particular country by applying the PCA method and a cluster analysis.

Air pollution from aviation is also discussed in the Transport Policy White Paper of the EU [42]. In 2017, almost 26% of the total greenhouse gas emissions in the EU-27 originated from the transport segment. The European Union appealed for a reduction of greenhouse gas emissions in the transport segment by 2030 at a level approximately 20% below that recorded in 2008.

The most significant increase in transport intensity has been observed in air transport. Despite improvements in fuel consumption, the aircraft emissions are expected to reach 7-fold or even 10-fold the 1990 value by 2050. This increase in greenhouse gas emissions from aviation and maritime transport was largely caused by an increase in transport intensity. The number of aviation passengers in the EU has tripled since 1993 [43].

**Figure 2:**
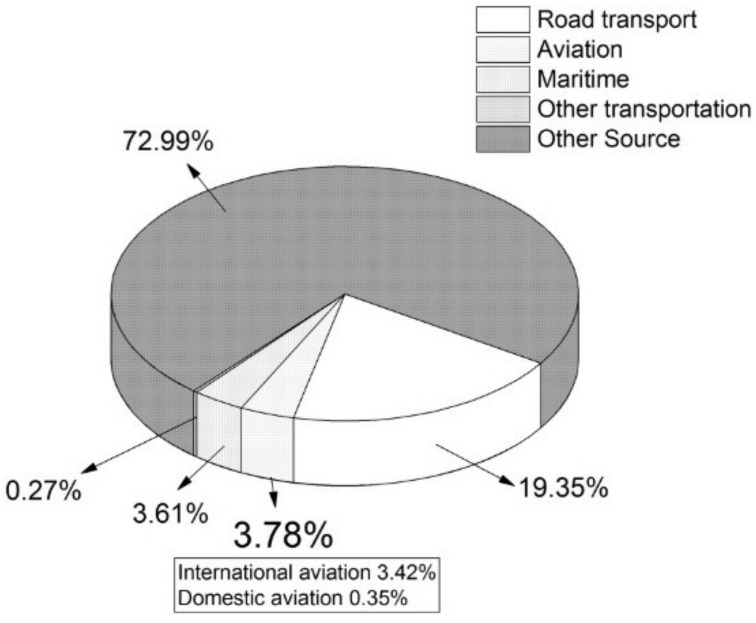
Percentage of aviation’s contribution to the total greenhouse gas emissions in the European Union [45].

The percentage of aviation’s contribution to the development of greenhouse gas emissions in the EU in 2017 is shown in Figure 2. In 2017, aviation (including international and domestic aviation) represented almost 4% of the total greenhouse gas emissions. Other sources of greenhouse gas emissions included the energy industry, industrial processes and product use, agriculture and waste management, excluding land use, land use change and forestry (LULUCF) [44].

**Figure 3:**
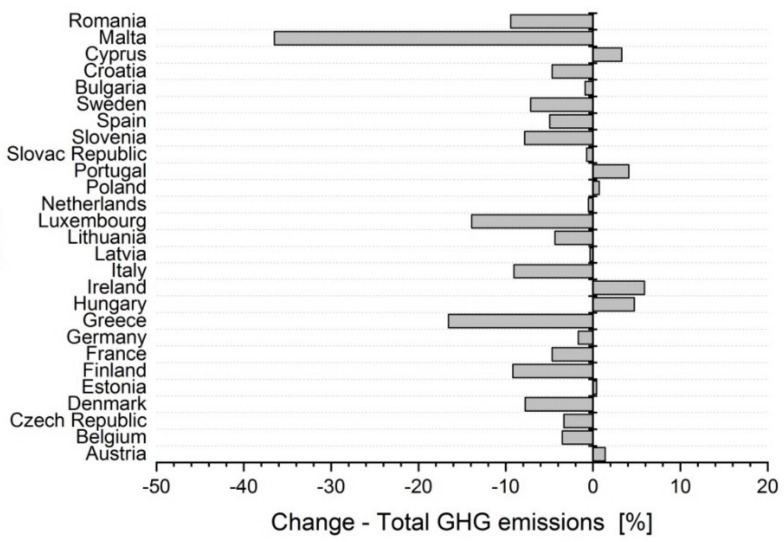
Change in the total greenhouse gas emissions (GHG emissions) (2012–2018) [46].

In individual EU member states, in the years 2012 to 2018, there were changes in the amount of the total greenhouse gas emissions (Figure 3). The greatest decreases, compared to the year 2012, were observed in Malta (−36%), Greece (−16%), Luxembourg (−14%), Italy (−9%) and Romania (−9%). On the opposite end of the spectrum, the greatest increases were observed in Ireland (+6%), Hungary (+5%) and Portugal (+4%). According to a report from the Commission to the European Parliament and the Council [45], many countries achieved such a reduction of emissions from the use of energy in buildings, from agriculture and from waste management. Emissions from industry and other sectors also decreased.

**Figure 4:**
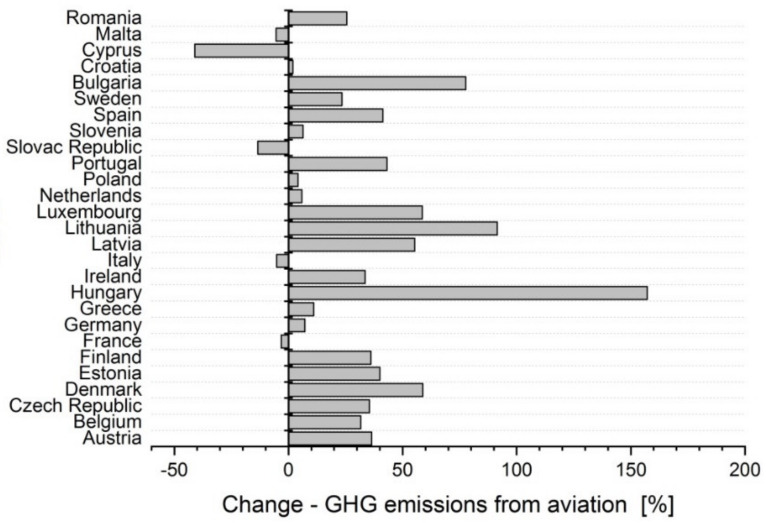
Change in the total greenhouse gas emissions (GHG emissions) from aviation (2012–2018) [46].

In the years 2012 to 2018, the total greenhouse gas emissions from aviation also exhibited some changes (Figure 4). In almost all EU-27 member states, there was an increase in the aviation greenhouse gas emissions. The largest increases, when compared to 2012, were observed in Hungary (+157%), Lithuania (+91%) and Bulgaria (+77%). The largest decreases were observed in Cyprus (–41%) and Slovakia (–14%).

**Figure 5:**
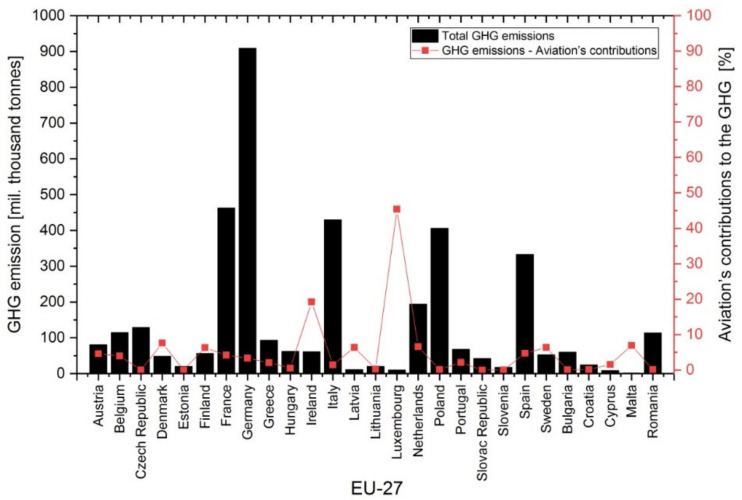
**Figure 5**. Greenhouse gas emissions (GHG emissions) and aviation’s contribution to GHG in the 27 European Union (EU-27) member states (2018) [46].

In 2018, out of all EU member states (Figure 5, column plot), the greenhouse gas emissions were at the highest level in Germany (almost 24% of the total value in the EU-27), followed by France (12% of the total value in the EU-27), Italy (11% of the total value in the EU-27), Poland (11% of the total value in the EU-27) and Spain (9% of the total value in the EU-27).

Aviation’s contribution to the production of greenhouse gas emissions in the EU-27 member states in 2018 are shown in Figure 5 (the line chart). High percentages of aviation greenhouse gas emissions were observed in Luxembourg (45%) and Ireland (20%). The percentages of aviation greenhouse gas emissions were similar in countries such as Denmark (8%), Malta (7%), Netherlands (7%), Sweden (almost 7%), Finland (6%) and Latvia (6%).

**Figure 6:**
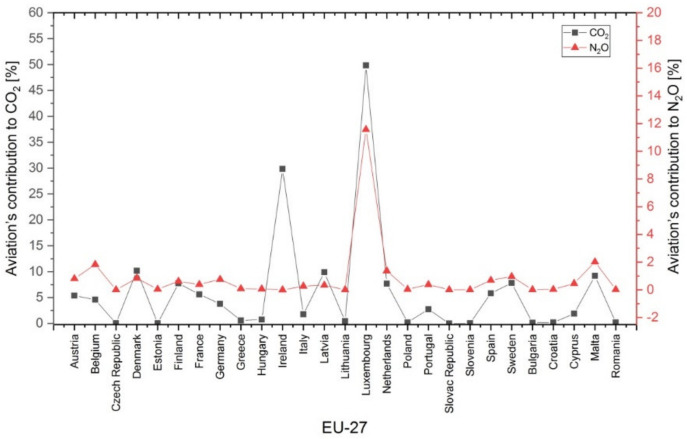
Percentage of aviation’s contribution to carbon dioxide (CO_2_) and nitrous oxide (N_2_O) emissions in the 27 European Union (EU-27) member states (2018, %) [47].

**Figure 7:**
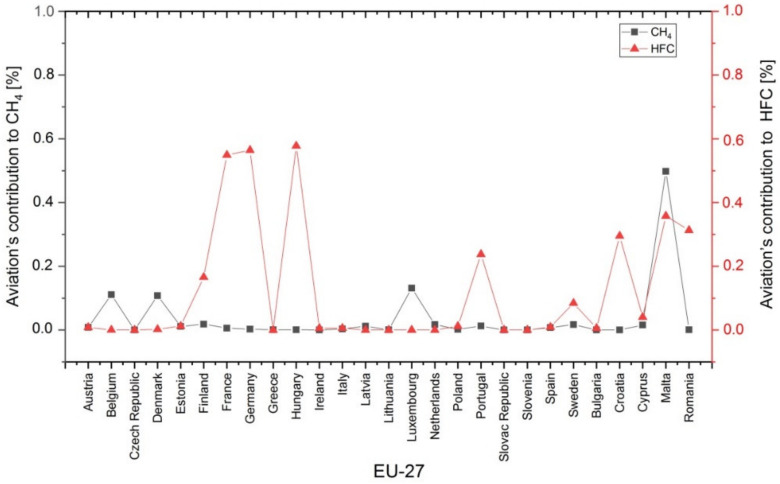
Percentage of aviation’s contribution to methane (CH4) and hydrofluorocarbons (HFC) emissions in the 27 European Union (EU-27) member states (2018, %) [47].

Figure 6 and Figure 7 represent the percentages of the 2018 aviation’s contribution to the total emissions of CO_2_, N_2_O, CH_4_ and HFC in individual EU-27 member states. The highest percentages of CO_2_ emissions from aviation (Figure 6, black plot) were observed in Luxembourg (almost 50%) and Ireland (30%). The highest percentage of N_2_O emissions from aviation (Figure 6, red plot) was observed in Luxembourg (approximately 12%).

In 2018, the percentages of aviation’s contribution to CH_4_ emissions (Figure 7, black plot) and HFC emissions (Figure 7, red plot) were below 0.6% in all EU countries.

### 3.2. Comparison of EU-27 Countries by Applying Multiple-Criteria Statistical Methods

The impact of aviation on greenhouse gas emissions in the EU member states was characterized using several selected parameters (Table 1). 

#### 3.2.1. PCA Method

When analyzing and evaluating the data, the first step was to identify whether the input variables correlated, i.e., whether they exhibited any relationships. As the monitored variables were measured in different units, the relationships between them were identified using the correlation matrix (Table 2). The relationship between two variables was determined by the coefficient of correlation *r.* We used the following scale: no correlation (|*r*| < 0.29), weak correlation (0.30 < |*r*| < 0.49), moderate correlation (0.50 < |*r*| < 0.79) and strong correlation (S, 0.80 < |*r*| < 1).

The results of the correlation matrix indicated that there were very strong correlations, for example, between aviation share of CO_2_ emissions (A11) and aviation share of total GHG emissions (A10) (r = 0.99), amount of aviation transported goods (A8) and aviation share of HFC emissions (A14) (r = 0.96), aviation share of total GHG emissions (A10) and aviation share of HFC emissions (A14) (r = 0.93), aviation share of CO_2_ emissions (A11) and aviation share of HFC emissions (A14) (r = 0.93), aviation share of total GHG emissions (A10) and amount of aviation transported goods (A8) (r = 0.90), aviation share of CO_2_ emissions (A11) and amount of aviation transported goods (A8) (r = 0.90), aviation share of CO_2_ emissions (A11) and number of aviation transported passengers (A7) (r = 0.86), aviation share of total GHG emissions (A10) and number of aviation transported passengers (A7) (r = 0.86), total greenhouse gas (A9) and number of aviation transported passengers (A7) (r = 0.86), total greenhouse gas (A9) and amount of aviation transported goods (A8) (r = 0.85) and number of commercial airports with more than 15,000 passengers per year (A6) and population (A1) (r = 0.87). Due to the fact that there were relatively strong relationships between many pairs, new independent variables were identified by applying the method of principal component analysis (PCA). This method facilitated replacing the original variables with new, independent variables—the principal components.

**Figure 8:**
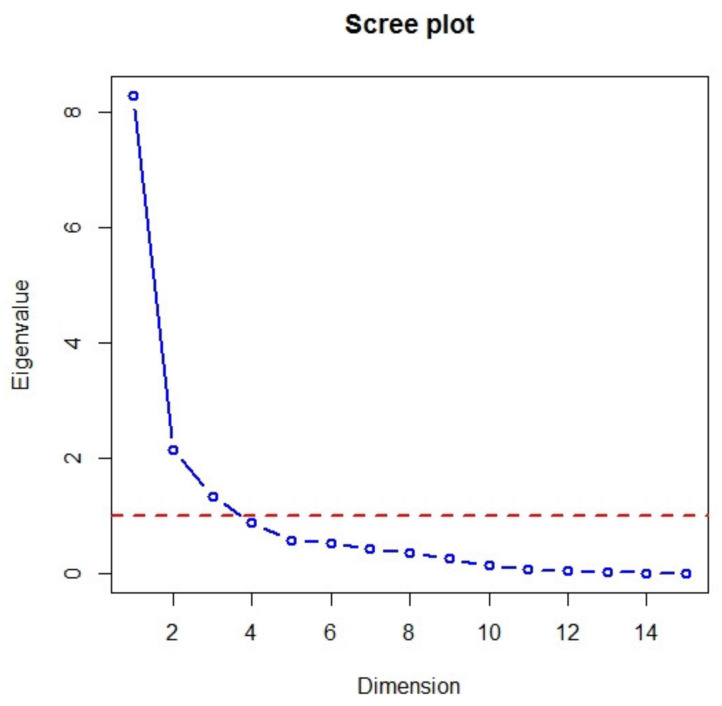
Scree plot for principal component analysis (Output: R Package).

The principal components were identified using the eigenvalues shown in Figure 8. A scree plot (Figure 8, blue line) displays the eigenvalues for each new component (dimension) in a downward curve. The red dashed line corresponds to an eigenvalue of 1. The eigenvalue for the first principal component was 8.3, the second was 2.1 and the third was 1.3 (Table 3). The first principal component described approximately 55.3% of the cumulative variance of the data, the second component described 14.3% and the third component described 8.9% of the cumulative variance. The other components represented approximately 21.5% of the cumulative variance. The eigenvalues and variance of the PCA components are listed in Table 3.

The number of principal components was identified by applying the Kaiser–Guttman rule. We considered the components with eigenvalues higher than 1. This means that the identification of new variables was carried out using the first three principal components, which together covered almost 78.5% of the cumulative variance in the data.

The eigenvector coefficients, i.e., the component matrix, for the first three principal components are listed in Table 4. The first principal component Dim1 consisted mainly of attributes A1, A2, A6 through A12, and A15. The second principal component Dim2 consisted mainly of attributes A3, A5 and A13. The third principal component Dim3 was mainly affected by attribute A4.

**Figure 9:**
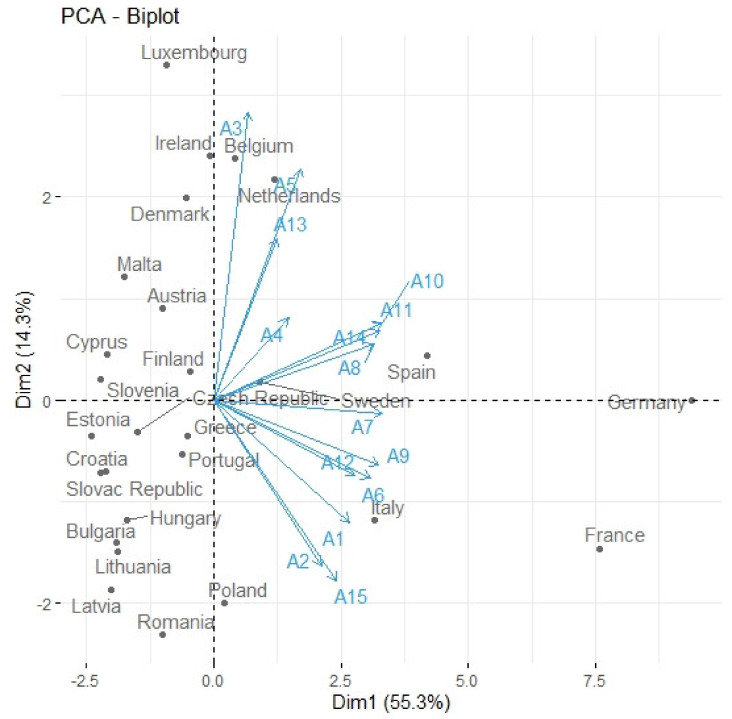
Biplot (Output: R Package), PCA-the principal component analysis, Dim1-the first principal component, Dim2-the second principal component, A1-population (million inhabitants), A2-area (km^2^), A3-gross domestic product per capita, A4-healthy life years (years), A5-life expectancy (years), A6-number of commercial airports with more than 15,000 passengers per year, A7-number of aviation transported passengers, A8-amount of aviation transported goods (tonnes), A9-total greenhouse gas (GHG) emissions (thousand tonnes), A10-aviation share of total GHG emissions (thousand tonnes), A11-aviation share of CO_2_ emissions (thousand tonnes), A12-aviation share of N_2_O emissions (thousand tonnes), A13-aviation share of CH_4_ emissions (thousand tonnes), A14-aviation share of HFC emissions (thousand tonnes), A15-amount of PM2.5 (tonnes).

The graphical representation of the data for the original attributes in the coordinate system of one principal component in relation to the second principal component is shown in Figure 9.

The plot indicates that France and Germany, when compared to other EU-27 member states, seem to be the outliers. Similarly, Luxembourg, Italy and Spain also exhibited marked differences from the other member states.

#### 3.2.2. Cluster Analysis

The member states with similar monitored characteristics were grouped by applying a cluster analysis. Agglomerative hierarchical methods were gradually applied: average linkage clustering, nearest neighbor algorithm, Ward’s method and median clustering. The distance measure was the Euclidean distance, which is the most frequently used measure, and the input variables were the three principal components created by applying the PCA method.

The verification of the accuracy of the results and the identification of the best clustering method were carried out using the cophenetic correlation coefficient CC (Table 5). The highest value of the cophenetic correlation coefficient determined the best clustering method. The closer its value is to one, the more suitable the method of hierarchical agglomerative clustering is for the expression of the structure of the analyzed data. The results indicated that the best clustering method was probably the average linkage method.

**Figure 10:**
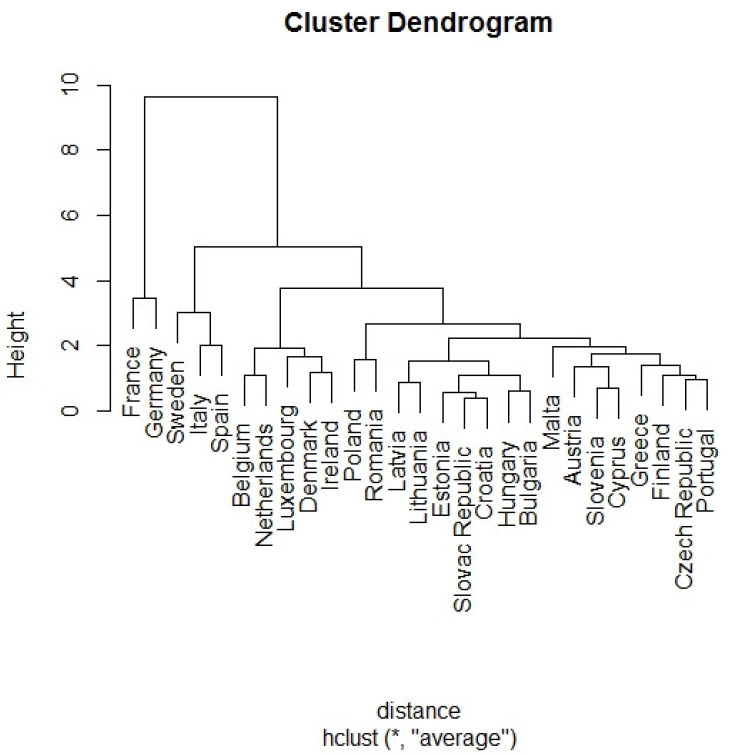
Dendrogram derived from the average linkage method (Output: R package).

The results of the cluster analysis of the selected parameters that characterized the countries and represented the impact of aviation on greenhouse gas emissions in the air of the EU-27 countries are shown in Figure 10. The dendrogram indicates that Germany and France formed one cluster. Another cluster consisted of Spain, Sweden and Italy. The third cluster consisted of Belgium, Netherlands, Luxembourg, Denmark and Ireland. The remaining member states were grouped into one big cluster (Table 6).

## 4. Conclusions

The purpose of the existing EU policies aimed at reducing air pollution is to achieve an air quality that does not cause significant negative effects on human health and the environment.

The total greenhouse gas emissions in the EU-27 have decreased in recent years, but the percentage of the contribution of all forms of transport (including the aviation) to the production of greenhouse gas emissions has increased.

The present study examined information on the greenhouse gas emissions in the EU-27 member states. The utilization of other available data (population, area, life expectancy, number of airports, etc.) facilitated a comparison of the individual member states by applying multiple-criteria statistical methods. The result was the creation of clusters consisting of similar member states, while also considering the impact of aviation on the amount of greenhouse gas emissions. The processed data indicated that the EU member states may be divided into four groups based on their similarities. Group 1 consisted of Germany and France; Group 2 consisted of Spain, Sweden and Italy; and Group 3 consisted of Belgium, Netherlands, Luxembourg, Denmark and Ireland. The remaining member states were grouped into one big cluster of countries with similar characteristics. This analysis of the impact of aviation on the amount of greenhouse gas emissions demonstrates the differences between individual EU countries.

Aviation is a strong sector within the European Union economy, and its importance exhibits continuing expansion. Nevertheless, every member state in the EU-27 should also realize that, along with the growth of air transport, the reduction of greenhouse gas emissions becomes increasingly important for the sake of public health and safety.

## Figures and Tables

**Table 1:** Selected attributes characterizing the 27 European Union (EU-27) member states (2018).

Attribute Designation	Attribute
A1	Population (million inhabitants)
A2	Area (km^2^)
A3	Gross domestic product per capita
A4	Healthy life years (years)
A5	Life expectancy (years)
A6	Number of commercial airports with more than 15,000 passengers per year
A7	Number of aviation transported passengers
A8	Amount of aviation transported goods (tonnes)
A9	Total greenhouse gas (GHG) emissions (thousand tonnes)
A10	Aviation share of total GHG emissions (thousand tonnes)
A11	Aviation share of CO_2_ emissions (thousand tonnes)
A12	Aviation share of N_2_O emissions (thousand tonnes)
A13	Aviation share of CH_4_ emissions (thousand tonnes)
A14	Aviation share of HFC emissions (thousand tonnes)
A15	Amount of PM2.5 (tonnes)

The analysis was carried out using the data obtained from the sources compiled by Eurostat (European Statistical Office), the European Environment Agency (EEA) and the OECD statistics (Organisation for Economic Co-operation and Development).

**Table 2:** Correlation matrix.

Variables	A1	A2	A3	A4	A5	A6	A7	A8	A9	A10	A11	A12	A13	A14	A15
A1	1														
A2	0.49	1													
A3	−0.08	−0.11	1												
A4	0.40	0.08	0.20	1											
A5	0.30	−0.05	0.55	0.45	1										
A6	**0.87**	0.56	−0.02	0.44	0.46	1									
A7	0.73	0.48	0.06	0.40	0.49	**0.83**	1								
A8	0.42	0.53	0.28	0.21	0.36	0.58	0.76	1							
A9	0.64	0.58	0.02	0.30	0.26	0.71	**0.86**	**0.85**	1						
A10	0.56	0.44	0.35	0.34	0.48	0.69	**0.86**	**0.90**	**0.82**	1					
A11	0.56	0.44	0.36	0.33	0.48	0.68	**0.86**	**0.90**	**0.82**	**0.99**	1				
A12	0.56	0.59	0.04	0.14	0.18	0.72	0.59	0.71	0.66	0.70	0.70	1			
A13	0.09	0.00	0.20	0.15	0.39	0.18	0.30	0.36	0.20	0.31	0.31	0.16	1		
A14	0.53	0.47	0.25	0.26	0.43	0.65	**0.83**	**0.96**	**0.83**	**0.93**	**0.93**	0.68	0.48	1	
A15	0.74	0.50	−0.21	0.24	0.09	0.70	0.69	0.42	0.75	0.44	0.44	0.55	0.13	0.42	1

Note: Bold values indicate a strong correlation (0.80 < |*r*| < 1), A1-population (million inhabitants), A2-area (km^2^), A3-gross domestic product per capita, A4-Healthy life years (years), A5-life expectancy (years), A6-number of commercial airports with more than 15,000 passengers per year, A7-number of aviation transported passengers, A8-amount of aviation transported goods (tonnes), A9-total greenhouse gas (GHG) emissions (thousand tonnes), A10-aviation share of total GHG emissions (thousand tonnes), A11-aviation share of CO_2_ emissions (thousand tonnes), A12-aviation share of N_2_O emissions (thousand tonnes), A13-aviation share of CH_4_ emissions (thousand tonnes), A14-aviation share of HFC emissions (thousand tonnes), A15-amount of PM2.5 (tonnes).

**Table 3:** Summary of the principal component analysis.

Component	1	2	3	4	5	6	7	8	9	10	11	12	13	14	15
Eigenvalue	8.3	2.1	1.3	0.87	0.57	0.53	0.41	0.36	0.25	0.12	0.06	0.03	0.01	0.002	0.0001
Variance (%)	55.3	14.3	8.9	5.8	3.8	3.5	2.7	2.4	1.7	0.83	0.40	0.23	0.11	0.013	0.0009
Cumulative Variance (%)	55.3	69.6	78.5	84.3	88.1	91.6	94.3	96.7	98.4	99.23	99.63	99.86	99.97	99.98	100.00

**Table 4:** Component matrix for the three principal components.

PCA	A1	A2	A3	A4	A5	A6	A7	A8	A9	A10	A11	A12	A13	A14	A15
Dim1	0.75	0.59	0.19	0.41	0.48	0.86	0.93	0.88	0.91	0.93	0.93	0.78	0.34	0.92	0.67
Dim2	−0.34	−0.46	0.79	0.23	0.64	−0.22	−0.04	0.15	−0.18	0.21	0.21	−0.21	0.48	0.19	−0.50
Dim3	0.41	−0.22	−0.05	0.67	0.40	0.32	0.12	−0.38	−0.10	−0.17	−0.18	−0.22	−0.05	−0.26	0.24

**Table 5:** Cophenetic correlation coefficient (CC) values.

Method	CC	Method	CC
Average linkage method	0.923	Nearest neighbor algorithm	0.902
Ward’s method	0.864	Median method	0.891

**Table 6:** Cluster analysis results.

Cluster	Countries
Cluster 1	France, Germany
Cluster 2	Sweden, Italy, Spain
Cluster 3	Belgium, Netherlands, Luxembourg, Denmark, Ireland
Cluster 4	Poland, Romania, Latvia, Lithuania, Estonia, Slovac Republic, Croatia, Hungary, Bulgaria, Malta, Austria, Slovenia, Cyprus, Greece, Finland, Czech Republic, Portugal

## References

[1] European Environment Agency (2019). Air Quality in Europe-2019 Report.

[2] Ragazzi M. (2017). Pollution and the Atmosphere: Designs for Reduced Emissions.

[3] Greenhouse Gas Emissionstatistics—Emissioninventories. https://ec.europa.eu/eurostat/statistics-explained/pdfscache/1180.pdf.

[4] United Nations Framework Convention on Climate Change—Declarations. https://eur-lex.europa.eu/eli/convention/1994/69/oj.

[5] Kyoto Protocol on Climate Change. https://eur-lex.europa.eu/legal-content/EN/TXT/HTML/?uri=LEGISSUM:l28060&from=SK.

[6] Directive 2003/87/EC of the European Parliament and of the Council of 13 October 2003 Establishing a Scheme for Greenhouse Gas Emission Allowance Trading within the Community and Amending Council Directive 96/61/EC. https://eur-lex.europa.eu/eli/dir/2003/87/oj.

[7] Directive 2008/50/EC of the European Parliament and of the Council of 21 May 2008 on ambient air quality and cleaner air for Europe. https://eur-lex.europa.eu/eli/dir/2008/50/oj.

[8] Baldauf R., Watkins N., Heist D., Bailey C., Rowley P., Shores R. (2009). Near-road air quality monitoring: Factors affecting network design and interpretation of data. Air Qual. Atmos. Health.

[9] Ren C., Tong S. (2008). Health effects of ambient air pollution–recent research development and contemporary methodological challenges. Environ. Health.

[10] Anderson H., Ponce de Leon A., Bland J., Bower J.S., Strachan D.P. (1996). Airpollution and daily mortality in London: 1987–92. BMJ.

[11] Burnett B., Brook J., Yung W., Dales R.E., Krewski D. (1997). Associationbetween ozone and hospitalization for respiratory diseasesin 16 Canadian cities. Environ. Res.

[12] Hoek G., Brunekreef B., Fischer P., Wijnen J.V. (2001). The associationbetween air pollution and heart failure, arrhythmia, embo-lism, thrombosis, and other cardiovascular causes of death ina time series study. Epidemiology.

[13] Mar T., Norris G., Koenig J., Larson T.V. (2000). Associations between airpollution and mortality in Phoenix, 1995–1997. Environ. Health Perspect..

[14] European Enviroment Agency (2015). Air quality in Europe-2015 Report.

[15] Skrucany T., Kendra M. (2020). Environmental assessment of selected impacts of the regional transport services. Svet. Dopravy.

[16] European Aviation Environmental Report 2019. https://ec.europa.eu/transport/sites/transport/files/2019-aviation-environmental-report.pdf.

[17] Koblen I., Szabo S., Krnacova K. (2013). Selected information on European Union research and development programmes and projects focused on reducing emissions from air transport. Naše More.

[18] Polishchuk V., Kelemen M., Gavurova B., Varotsos C., Andoga R., Gera M., Christodoulakis J., Sousek R., Kozuba J., Blistan P. (2019). Correction: A Fuzzy Model of Risk Assessment for Environmental Start-Up Projects in the Air Transport Sector. Int. J. Environ. Res. Public Health.

[19] Koscak P., Berezny S., Vajdova I., Koblen I., Ojciec M., Matiskova M., Puskas T. (2020). Reducing the Negative Environmental Impact of Winter Airport Maintenance through Its Model Design and Simulation. Int. J. Environ. Res. Public Health.

[20] Jakubiak M. (2015). Environmental impact of air transport-Case study of Krakow Airport. Logistyka.

[21] Cerro J.C., Cerdà V., Querol X., Alastuey A., Bujosa C., Pey J. (2020). Variability of air pollutants, and PM composition and sources at a regional background site in the Balearic Islands: Review of western Mediterranean phenomenology from a 3-year study. Sci. Total Environ..

[22] Mocerino L., Murena F., Quaranta F., Toscano D. (2020). A methodology for the design of an effective air quality monitoring network in port areas. Sci. Rep..

[23] Deshmukh P., Kimbrough S., Krabbe S., Logan R., Isakov V., Baldauf R. (2020). Identifying air pollution source impacts in urban communities using mobile monitoring. Sci. Total Environ..

[24] Dong X., Zhao X., Peng F., Wang D. (2020). Population based Air Pollution Exposure and its influence factors by Integrating Air Dispersion Modeling with GIS Spatial Analysis. Sci. Rep..

[25] Vichi F., Frattoni M., Imperiali A., Balducci C., Cecinato A., Perilli M., Romagnoli P. (2016). Civil aviation impacts on local air quality: A survey inside two international airports in central Italy. Atmos. Environ..

[26] Carslaw D.C., Beevers S.D., Ropkins K., Bell M.C. (2006). Detecting and quantifying aircraft and other on-airport contributions to ambient nitrogen oxides in the vicinity of a large international airport. Atmos. Environ..

[27] Wilkerson J.T., Jacobson M.Z., Malwitz A., Balasubramanian S., Wayseon R., Naiman A.D., Lele S.K. (2010). Analysis of emission data from global commercial aviation: 2004 and 2006. Atmos. Chem. Phys..

[28] Ivković I., Čokorilo O., Kaplanović S. (2018). The estimation of GHG emission costs in road and air transport sector: Case study of Serbia. Transport.

[29] Peeters J.H.A.M. (1998). Aviation and air pollution. Stud. Environ. Sci..

[30] Vasilaki V., Volcke E.I.P., Nandi A.K., Loosdrecht M.C.M., Katsou E. (2018). Relating N_2_O emissions during biological nitrogen removal with operating conditions using multivariate statistical techniques. Water Res..

[31] Bellandi G., Weijers S., Gori R., Nopens I. (2020). Towards an online mitigation strategy for N_2_O emissions through principal components analysis and clustering techniques. J. Environ. Manag..

[32] Miranda I.N.G., Macías F.V., Mesa G.A.P. (2020). Application of multivariate methods and geoestatistics to model the relationship between CO2 emissions and physicochemical variables in the hidrosogamoso reservoir, Colombi. Acta Limnol. Bras..

[33] Ni X., Wang H., Che C., Hong J., Sun Z. (2019). Civil aviation safety evaluation based on deep belief network and principal component analysis. Saf. Sci..

[34] Yang Y., Wu W., Fang P. (2018). Comprehensive Evaluation Approach to Aviation Maintenance Support Ability Based on PCA. Adv. Intell. Syst. Comput..

[35] Azid A., Juahir H., Toriman M.E., Kamarudin M.K.A., Saudi A.S.M., Hasnam C.N.C., Aziz N.A.A., Azaman F., Latif M.T., Zainuddin S.F.M. (2014). Prediction of the level of air pollution using principal component analysis and artificial neural network techniques: A case study in Malaysia. Water Air Soil Pollut..

[36] Kwon Y., Lee H., Lee H. (2018). Implication of the cluster analysis using greenhouse gas emissions of Asian countries to climate change mitigation. Mitig. Adapt. Strat. Glob. Chang..

[37] Map of EU Countries after Brexit. https://maproom.net/shop/eu-map/.

[38] Johnson R.A., Wichern D.W. (1992). Applied Multivariate Statistical Analysis.

[39] Jolliffe I.T. (2002). Principal Component Analysis and Factor Analysis, Chap 7. Principal Component Analysis.

[40] Everitt B.S., Landau S., Leese M. (2001). Cluster Analysis.

[41] Hartigan J.A. (1975). Clustering Algorithms.

[42] White Paper. https://eurlex.europa.eu/LexUriServ/LexUriServ.do?uri=COM:2011:0144:FIN:EN:PDF.

[43] Greenhouse Gas Emission from Transport in Europe. https://www.eea.europa.eu/data-and-maps/indicators/transport-emissions-of-greenhouse-gases/transport-emissions-of-greenhouse-gases-12.

[44] Emissions from Planes and Ships: Facts and Figures. https://www.europarl.europa.eu/news/en/headlines/priorities/climate-change/20191129STO67756/emissions-from-planes-and-ships-facts-and-figures-infographic.

[45] European Commission (2018). Report from the Commission to the European Parliament and the Council EU and the Paris Climate Agreement: Taking Stock of Progress at Katowice COP.

[46] Eurostat. https://ec.europa.eu/eurostat/web/climate-change/data/database.

[47] OECD Statistics. https://stats.oecd.org/.

